# Plasma-derived small extracellular vesicles unleash the angiogenic potential in head and neck cancer patients

**DOI:** 10.1186/s10020-023-00659-w

**Published:** 2023-05-24

**Authors:** Luisa Tengler, Julia Schütz, Moritz Tiedtke, Jadwiga Jablonska, Marie-Nicole Theodoraki, Katja Nitschke, Christel Weiß, Elena Seiz, Annette Affolter, Frederic Jungbauer, Anne Lammert, Nicole Rotter, Sonja Ludwig

**Affiliations:** 1 Department of Otorhinolaryngology, Head and Neck Surgery, Medical Faculty Mannheim, University Hospital Mannheim, University of Heidelberg, Mannheim, Germany; 2 Translational Oncology, Department of Otorhinolaryngology, University Hospital Essen, University of Duisburg-Essen, Essen, Germany; 3 Department of Otorhinolaryngology, Head and Neck Surgery, Ulm University Medical Center, Ulm, Germany; 4 Department of Urology and Urosurgery, Medical Faculty Mannheim, University Hospital Mannheim, University of Heidelberg, Mannheim, Germany; 5 Department of Medical Statistics and Biomathematics, Medical Faculty Mannheim, University Hospital Mannheim, University of Heidelberg, Mannheim, Germany

**Keywords:** Small extracellular vesicles, Exosomes, Angiogenesis, Head and neck cancer, Plasma, Endothelial cells

## Abstract

**Background:**

In Head and neck cancer (HNC) angiogenesis is essential for tumor progression and metastasis. Small extracellular vesicles (sEVs) from HNC cell lines alter endothelial cell (EC) functions towards a pro-angiogenic phenotype. However, the role of plasma sEVs retrieved from HNC patients in this process is not clear so far.

**Methods:**

Plasma sEVs were isolated on size exclusion chromatography columns from 32 HNC patients (early-stage UICC I/II: 8, advanced-stage UICC III/IV: 24), 12 patients with no evident disease after therapy (NED) and 16 healthy donors (HD). Briefly, sEVs were characterized by transmission electron microscopy (TEM), nanoparticle tracking analysis (NTA), BCA protein assays and Western blots. Levels of angiogenesis-associated proteins were determined using antibody arrays. The interaction of fluorescently-labeled sEVs with human umbilical vein ECs was visualized by confocal microscopy. The functional effect of sEVs on tubulogenesis, migration, proliferation and apoptosis of ECs was assessed.

**Results:**

The internalization of sEVs by ECs was visualized using confocal microscopy. Based on antibody arrays, all plasma sEVs were enriched in anti-angiogenic proteins. HNC sEVs contained more pro-angiogenic MMP-9 and anti-angiogenic proteins (Serpin F1) than HD sEVs. Interestingly, a strong inhibition of EC function was observed for sEVs from early-stage HNC, NED and HD. In contrast, sEVs from advanced-stage HNC showed a significantly increased tubulogenesis, migration and proliferation and induced less apoptosis in ECs than sEVs from HD.

**Conclusions:**

In general, plasma sEVs carry a predominantly anti-angiogenic protein cargo and suppress the angiogenic properties of ECs, while sEVs from (advanced-stage) HNC patients induce angiogenesis compared to HD sEVs. Thus, tumor-derived sEVs within the plasma of HNC patients might shift the angiogenic switch towards angiogenesis.

**Supplementary Information:**

The online version contains supplementary material available at 10.1186/s10020-023-00659-w.

## Background

Head and neck cancers (HNCs) are among the most common tumor entities worldwide (Sung et al. 2021). Frequent tumor locations are the oral cavity, oropharynx and larynx with the majority being Head and Neck squamous cell carcinomas (HNSCC). HNCs are usually advanced at the time of diagnosis, therefore often elude standard oncologic treatments, which results in tumor persistence or recurrence (Johnson et al. 2020). Novel therapeutics, such as anti-PD-1 immunotherapy, have fallen short of expectations with low therapeutic response rates, limiting patient prognosis (Ferris et al. 2016; Whiteside 2018). The identification of mechanisms critical for tumor evasion is urgently needed in order to provide effective therapy and improve the prognosis of HNC patients.

A key feature of solid tumors such as HNCs is their ability to induce angiogenesis. Tumor angiogenesis involves the formation and sprouting of new blood vessels from the vasculature to ensure the supply of oxygen and vital nutrients, especially in advanced tumors (Adams and Alitalo 2007). Angiogenesis is dependent on endothelial cell (ECs) proliferation, migration, and tube formation. While in healthy tissues pro- and anti-angiogenic factors are balanced, in the tumor microenvironment pro-angiogenic factors dominate considerably. One of the crucial factors that trigger the angiogenic switch is the hypoxia created by the growing tumor mass. In this context the expression of the hypoxia-induced factor-1a (HIF-1a) transcription factor is increased, which in turn activates several pro-angiogenic factors, such as vascular endothelial growth factor (VEGF) and matrix metalloproteinase 9 (MMP-9) (Bergers and Benjamin 2003).sEVs are nanosized lipid vesicles (30–120 nm) with an endosomal origin and belong to the group of extracellular vesicles (EVs). sEVs are crucial mediators of intercellular communication and can mediate various signaling pathways in the tumor microenvironment (TME). Recently, we have shown that sEVs have a suppressive effect on almost all immune cells, including T cells and NK cells, and serve as a surrogate marker for active HNCs (Ludwig et al. 2017). So far, tumor-derived sEVs (TEV) from HNC cell lines have been reported to contain pro-angiogenic proteins and nucleic acids that stimulate endothelial cells in their proliferation, migration, and ultimately blood vessel formation (Ludwig et al. 2018). Together with other TME-related factors, such as hypoxia and enhanced energy metabolism, angiogenesis provides a good foundation for tumor progression.

So far, little is known about the role of plasma sEVs in angiogenesis. This study provides a deeper insight in the angiogenic potential of sEVs and their potential as diagnostic and prognostic value in HNC.

## Materials and methods

### Patients

Venous blood was collected from head and neck cancer (HNC, n = 32) as well as no evident disease patients (NED, n = 12) visiting the Department of Otorhinolaryngology, Head and Neck Surgery of the University Hospital Mannheim from 2019 to 2022 and from healthy donors (HD, n = 16). The HNC patients were exclusively patients with squamous cell carcinoma, which will be referred to as HNC patients for simplicity. The study was approved by the local ethics committee (2021-552 und 2019-697N) and all participants signed informed consent before study inclusion. For plasma separation, the blood samples were centrifuged at 2000 g for 10 min at room temperature (RT), aliquoted and frozen at − 80 °C.

### Size-exclusion chromatography

sEVs from plasma were prepared by size exclusion chromatography as previously described by Hong et al. (2016): Briefly, freshly thawed plasma specimens were differentially centrifuged at 2000*g* for 10 min at RT and at 14,000*g* for 30 min at 4 °C, followed by ultrafiltration (Millipore filter, 0.22 µm). Self-made mini-size-exclusion chromatography columns with 10 mL Sepharose CL-2B (Cytiva, #17-0140-01) gel volume were prepared and 1 mL plasma was loaded and eluted with phosphate-buffered saline (PBS) to retrieve 1 mL fractions. The 4th fraction was collected and used for further studies.

### *BCAprotein assay*

The protein content of sEVs was analyzed using Pierce™ BCA protein assay (Thermo Fisher Scientific) according to the manufacturer’s instructions.

### Nanoparticle tracking analysis (NTA)

Nanoparticle tracking analysis (NTA) was performed on ZetaView® TWIN (Particle Metrix) to determine the size distribution and concentration of the isolated particles. Freshly isolated plasma sEVs from HNC patients were diluted up to 1:10,000 in PBS and from HD and NED up to 1:2000 in PBS and measured at room temperature (RT). Concentration and size ranges were calculated by ZetaView Software (Particle Metrix Version 8.05.11 SP1 and SP2, Sensitivity 80%, Shutter 100, 11 positions, 2 cycles).

### Transmission electron microscopy

Transmission electron microscopy (TEM) of plasma sEVs was performed at the Electron Microscopy Core Facility of Heidelberg University as described previously (Jablonska et al. 2022). For negative staining, freshly prepared sEVs were allowed to adsorb to formvar/carbon-coated copper grids and stained with 3% uranyl acetate. Micrographs were captured using a JEM1400 transmission electron microscope (JEOL Ltd) with a bottom-mounted 4K CMOS camera (TemCam F416; TVIPS).

### Western blots

Plasma sEVs (10 μg) in non-reducing (CD63 only) or reducing sample buffer were separated on 4–20% polyacrylamide gels (Bio-Rad, #4561094) and transferred onto nitrocellulose membranes (Bio-Rad, #1620090). Briefly after blocking, the membrane was incubated with the following primary antibodies overnight at 4 °C: anti-CD63 (Invitrogen, #10628D, 1:250), anti-CD9 (Invitrogen, #10626D, 1:500), anti-TSG101 (Invitrogen, #PA5-31260; 1:500), anti-Grp94 (CST, #2104; 1:1000 in 5% BSA in PBS), anti-ApoA1 (CST, #3350; 1:1000). After washing, HRP-conjugated secondary antibodies (IgG Rabbit anti-Mouse, Invitrogen, #31450, 1:10,000 or IgG Goat anti-Rabbit, Invitrogen, #31460, 1:10,000) were added and incubated for 1 h at RT. The chemiluminescence signal was elicited by SuperSignal™ West Dura™ Chemiluminescence Substrate (Thermo Scientific, #34076) according to the manufacturer’s instructions.

### Angiogenesis antibody arrays

The relative levels of human angiogenesis-related proteins in sEVs were measured using the Human Angiogenesis Array Kit (R&D Systems Inc.). sEVs (140 µg protein) from 4 HNC patients and 3 HDs were incubated with the array according to the manufacturer’s instructions, and the results were analyzed using Image J.

### Cell culture

Human umbilical vein cells (HUVEC) were obtained from Gibco (#C0035C, 1 female/ Lot#2044494 and 1 male newborn/Lot#2278504) and cultured in Human Large Vessel Endothelial Cell Basal Medium (Gibco, #M-200–500) with 2% (v/v) Large Vessel Endothelial Supplement (LVES; Gibco, #A14608-01) at 37 °C and 5% CO_2_ up to passage 6. For functional assays, LVES was replaced by 2% EV-depleted FBS (Gibco, #A2720801).

### Cell proliferation assay

HUVECs (5,000 cells) were co-incubated with 5 µg sEVs per well in a 96-well plate for 24 h. As controls, LVES-containing medium (2% LVES) or PBS (CTRL) was used. MTS cell proliferation assay was performed according to the manufacturer’s instructions (Abcam, #ab197010). Briefly, the MTS reagent was added at a dilution of 1:10 and incubated for 3 h. Absorbance was measured at 490 nm with a Tecan Infinite® 200 PRO microplate reader.

### Endothelial tube formation

HUVECs (20,000) were placed on top of 100 µL Geltrex® LDEV-Free Reduced Growth Factor Basement Membrane Matrix (Gibco, #A14132-02) in 48-well plates and co-incubated with 10 µg sEVs, LVES or PBS (CTRL). After 8 h, tubules were imaged, using phase contrast microscopy at 2.5 × magnification (Axiovert 25 CFL, Carl Zeiss Microscopy). For better visualization, tubules were stained with 2 µg/mL Calcein AM (Invitrogen, #L3224) for 30 min at 37 °C. Tubule length and the number of meshes were analyzed with the Angiogenesis Analyzer developed for the Image J software (Carpentier 2012).

### Wound healing

HUVECs were grown to confluence in wells of 48-well plates. Monolayers were scratched using pipet tips and cells were co-incubated in the presence of sEVs (10 µg per well). As controls, PBS (CTRL) or 2% LVES-containing medium was added. After 3-, 6-, 12- and 24-h incubation, the wound was imaged using an Axiovert 25 CFL inverted microscope at 10 × magnification. After completion of the assay, HUVECs were fixed with 4% paraformaldehyde, stained with 0.5% crystal violet in distilled water, and subsequently imaged again. Wound closure was analyzed using ImageJ and results are expressed as a percentage of the recovery.

### Apoptosis assay

To assess apoptosis of ECs Caspase-Glo® 3/7 (Promega, #G8091) assays were performed. HUVECs were seeded in 96-well-plates at a density of 5,000 cells/well in 100 µL. The cells were immediately treated with 50 µL sEVs (5 µg) in PBS and after 24 h an equal volume of luminogenic caspase-3/7 substrate was added and incubated at RT for 1 h. Luminescence was read with a Tecan Infinite® 200 PRO microplate reader. As controls, cells were seeded in an LVES-containing medium or treated with PBS (CTRL).

### sEV internalization

Isolated sEVs were labeled with PKH67 membrane-labeling solution (Sigma-Aldrich, #PKH67GL-1KT) according to the manufacturer’s instructions. Briefly, 600 µg/ml sEV solution was incubated with 2 µM PKH67 solution for 5 min at RT to stain the vesicles’ membrane. Excessive dye was removed by Spin Columns (Invitrogen, #4484449). HUVECs (20,000) were seeded on 8-well-chambered slides (Ibidi, #80826). After 24 h, 10 µg labeled sEVs, labeled PBS or PBS only were incubated in a serum-free medium for 4 h. To wash off sEVs bound to the HUVEC surface, cells were treated with stripping buffer (14.6 g NaCl, 2.5 mL acetic acid, 500 mL distilled water) for 2 min, followed by extensive washing. Cells were fixed with 4% paraformaldehyde for 20 min at RT, permeabilized with 0.1% Triton X-100 for 3 min and labeled with Phallodin-iFluor 647 (1:500) and DAPI (1:500) for 1 h. Imaging was performed using a Leica SP5 MP confocal microscope (Leica Microsystems) and the images were processed using ImageJ software. Imaging, processing, and analysis were done under constant settings across all samples.

### Statistical analysis

Results were graphed and analyzed using GraphPad Prism (9.4.1) and SAS software (release 9.4), respectively. Kruskal–Wallis tests were used to compare groups regarding non-normally distributed outcomes and ANOVA tests for normally distributed outcomes. In the case of a significant test result post hoc tests have been conducted for pairwise comparisons. Because of the small sample sizes, p values have not been corrected for multiple testing (i.e. Bonferroni correction). Quantitative data is presented as mean ± SD (standard deviation) unless otherwise stated. In general, the result of a statistical test has been considered as statistically significant for p less than 0.05. Statistically significant outliers (with p < 0.01) detected with the Grubbs’ test were excluded.

## Results

### Clinicopathological characterization of the study participants

The clinicopathological characteristics of patients are summarized in Table 1. In this study, we analyzed 32 HNC patients, 12 patients with no evident disease (NED) and 16 healthy donors (HD). The study cohort was representative of HNC patients with predominantly male patients and a median age of 64 years. HDs were age- (median age 54 years) and gender-matched (56% male, 44% female). All patients and HDs were Caucasian. The tumor subsets were in decreasing frequency in the oropharynx, oral cavity, larynx, and hypopharynx. Infection with human papillomavirus, detected by p16 positivity in immunohistochemistry, was prevalent in 39% of tested HNC patients. Regarding other risk factors, the majority of patients smoked tobacco (82%) and/or consumed alcohol (71%). In this study, about half of the patients had large tumors (T3/4). Most tumors spread to lymph nodes (N > 0: 70%) with no evidence of distant metastases (M0: 100%) at the time of diagnosis. According to the 8^th^ edition of UICC classification, most patients were diagnosed with advanced stage HNC (UICC III/IV: 70%). Therapy regimens of patients comprised surgery (16%), surgery with adjuvant (chemo)radiation (59%), primary chemoradiation (18%) and palliative radiotherapy (7%).

**Table 1:** Clinicopathological characteristics of the patients

	HNC (n = 32)		NED (n = 12)			All (n = 44)	
Age							
Median	62.5		66			64	
	n	%	n		%	n	%
Sex							
Male	20	62.5	6		50	26	59.1
Female	12	37.5	6		50	18	40.9
Tumor site							
Oropharynx	19	59.4	5		41.7	24	54.5
Oral cavity	6	18.8	1		8.3	7	15.9
Larynx	3	9.4	5		41.7	8	18.2
Hypopharynx	4	12.5	1		8.3	5	11.4
HPV status^+^							
Negative	18	56.3	1		8.3	19	43.2
Positive	8	25.0	4		33.0	12	27.3
n/a	6	18.8	7		58.3	13	29.5
Smoking							
Never	3	9.4	1		8.3	4	9.1
Former	9	28.1	4		33.3	13	29.5
Current	19	59.4	4		33.3	23	52.3
n/a	1	3.1	3		25.0	4	9.1
Alcohol consumption							
Never	4	8.8	0		0	4	9.1
Former	8	26.5	6		50.0	14	31.8
Current	15	44.1	1		8.3	16	36.4
n/a	5	20.6	5		41.7	10	22.7
Tumor size							
T1	2	6.3		4	33.3	6	13.6
T2	14	43.8		4	33.3	18	40.9
T3	9	28.1		1	8.3	10	22.7
T4	7	21.9		3	25.0	10	22.7
Lymph node status							
N0	9	28.1		4	33.3	13	29.5
N1	8	25.0		5	41.7	13	29.5
N2	9	28.1		2	16.7	11	25.0
N3	6	18.8		1	8.3	7	15.9
Metastases							
M0	32	100		12	100	44	100
UICC stages^*^							
I	4	12.5		3	25.0	7	15.9
II	4	12.5		2	16.7	6	13.6
III	9	28.1		4	33.3	13	29.5
IV	15	46.9		3	25.0	18	40.9
Therapy							
Surgery	4	12.5		3	25.0	7	15.9
Surgery plus CRT	14	43.8		5	41.7	19	43.2
Surgery plus RT	6	18.8		1	8.3	7	15.9
Primary CRT	5	15.6		3	25.0	8	18.2
Palliative RT	3	9.4		0	0	3	6.8
Time between end of therapy and sample collection							
< 6 months				3	25.0		
> 6 months				3	25.0		
> 2 years				6	50.0		

*n* number of patients, *CRT* chemoradiotherapy, *RT* radiotherapy, ^+^p16 positivity, *International Union Against Cancer (UICC) classification 8th edition

### Characterization of sEVs

The isolated plasma sEVs were characterized for their morphology by transmission electron microscopy (TEM), for the particle concentration and size distribution by nanoparticle tracking analysis (NTA), and for the particle concentration and protein content by BCA assay and Western blots, respectively.

**Fig. 1:**
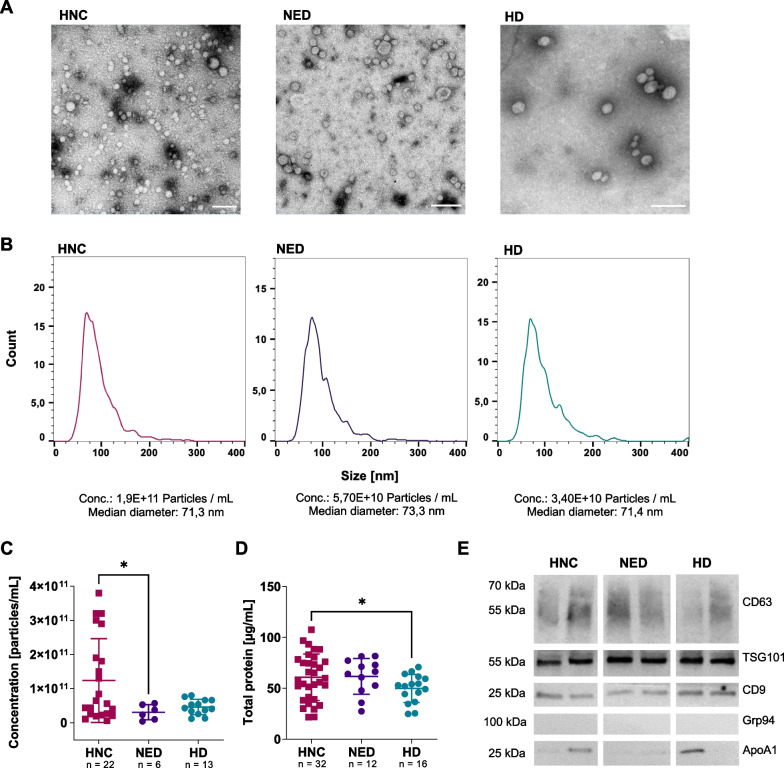
Characterization of plasma sEVs. sEVs were freshly isolated from plasma of HNC, NED patients and HD by size-exclusion chromatography and characterized for their morphology, particle concentration and protein content. **A** Representative TEM images of sEVs from HNC, NED patients and HD are shown (magnification = 25.000x, scale = 100 nm). **B** Size distribution, concentration and median diameter of the particles were detected using NTA. C NTA particle concentration of HNC, NED patients and HD were visualized by scatter plots. D Comparison of total protein content in 1 mL of isolated sEVs measured by BCA protein assays. E Western blots were performed using 10 μg protein and the presence of typical vesicle proteins CD9, CD63 and TSG101 and the absence of contaminating proteins (Grp94, ApoA1) were shown. P values below 0.05 were considered as significant (*p < 0.05)

TEM confirmed a spherical morphology of the isolated sEVs (Fig. 1A). Using NTA, sEVs from HNC patients (1.24 × 10^11^ particles/mL) revealed higher concentrations than NED and HD sEVs (NED: 3.11 × 10^10^, HD: 4.62 × 10^10^ particles/mL), while median sizes were comparable (Fig. 1B, C). Protein levels of sEVs from HNC (60.8 ± 22.9 µg/mL) and NED (61.6 ± 17.6 µg/mL) were elevated compared to HD (49.8 ± 13.8 µg/mL) (Fig. 1D).

Western blots confirmed the presence of vesicle-associated proteins, i.e. the tetraspanins (CD9 and CD63), and TSG101. Besides, the absence of cellular components such as Grp94 was confirmed and lipoproteins like ApoA1 were shown to be reduced (Fig. 1E, Additional file 2: Fig. S1).

### Plasma sEVs contain pro- and anti-angiogenic proteins

**Fig. 2:**
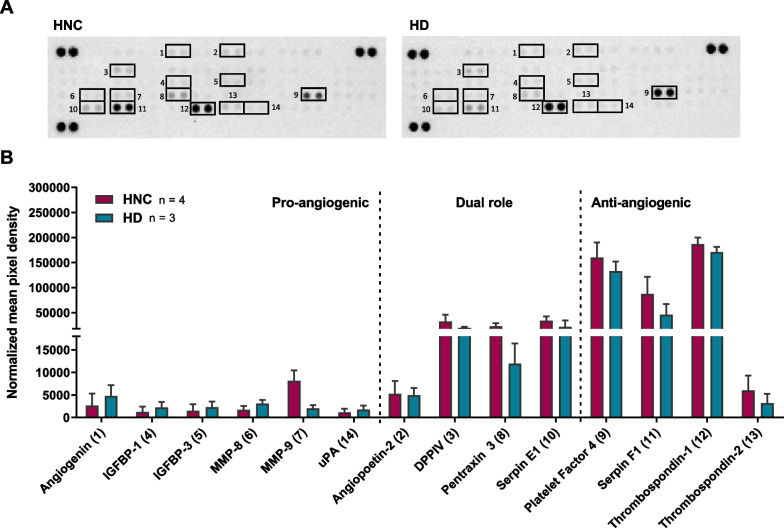
Relative content of angiogenesis-associated proteins. The angiogenesis antibody arrays were used to measure the angiogenic profile of 140 μg total protein of the prepared sEVs from HNC patients and healthy donors. The pixel density of each spot was evaluated using ImageJ. **A** Representative images of angiogenesis antibody arrays of one HNC patient and one HD are shown. **B** Column bar graph summarizes the protein levels of angiogenesis-associated markers of sEVs from HNC patients and HDs. Data are shown as means ± SEM (standard error of the mean)

To reveal the angiogenic protein profiles of the sEVs, sEV proteomes from HNC patients and HDs were assessed using antibody arrays and compared for their pro- and anti-angiogenic features (Fig. 2).

Overall, antibody arrays showed enrichment in anti-angiogenic proteins, such as platelet factor 4, serpin F1 and thrombospondin-1, compared to pro-angiogenic proteins in all plasma sEVs (Fig. 2B). Furthermore, HNC sEVs contain four times more pro-angiogenic MMP-9, double the amount of dual-function pentraxin-3 and dipeptidyl peptidase IV (DPPIV) is increased by 63% compared to HD sEVs. Besides, anti-angiogenic serpin F1 is more than twice as high in HNC compared to HD sEVs (Additional file 1: Table S1).

### Plasma sEVs are internalized by HUVECs and modulate their function

**Fig. 3:**
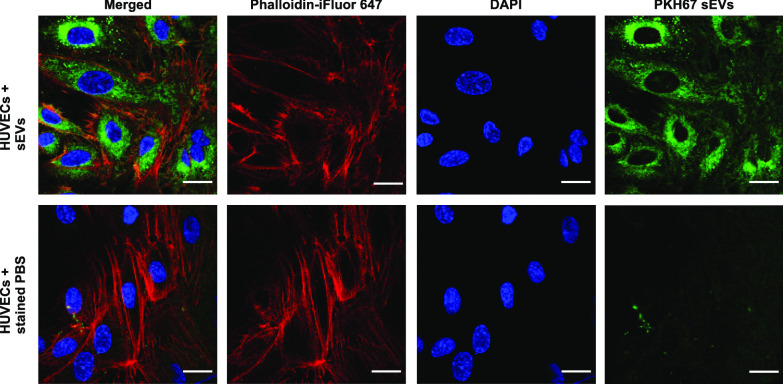
Plasma sEVs are internalized by HUVECs. Confocal microscopy of HUVECs after 4 h incubation with 20 µg PKH67-stained sEVs (upper row) or stained PBS as control (lower row). Magnification = 40x, scale = 20 µm

**Fig. 4:**
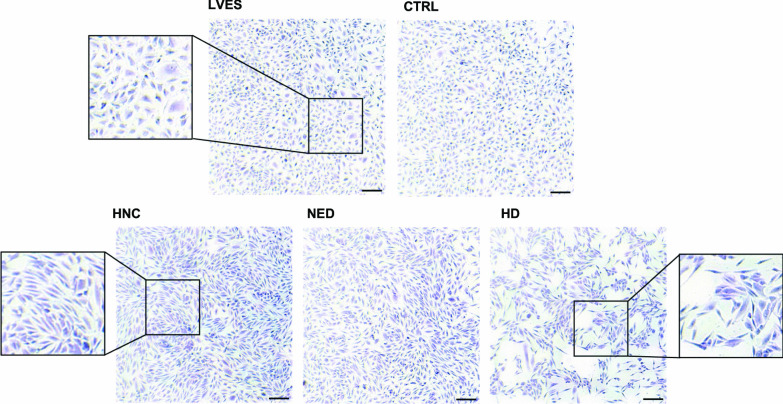
Crystal violet staining of HUVECs co-incubated with sEVs. Crystal violet staining of HUVECs after 24 h of stimulation with sEVs from HNC, NED patients and HD. HUVECs co-incubated with sEVs from HNC patients showed a rather elongated morphology and remained confluent compared to HUVECs with HD sEVs. The control images, HUVECs with PBS (CTRL) and LVES, visualized cobblestone-shaped and attached cells. Magnification = 2.5x, scale = 200 µm

To visualize the interaction of the isolated sEVs with ECs, sEVs were dyed with PKH67, incubated with human umbilical vein endothelial cells (HUVECs) and confocal microscopy was performed after 4 h. As a control, identically stained PBS without sEVs was used (Fig. 3). Internalization of PKH67-stained vesicles by ECs was observed after only 4 h. To analyze the effect of sEVs on the EC function, vesicles from HNC, NED patients or HDs were incubated with HUVECs. Large vessel endothelial supplement (LVES), which contains pro-angiogenic factors like epidermal growth factor (EGF) and basic fibroblast growth factor (bFGF), was used as a positive control and PBS as a negative control (Fig. 4). The effective amount of sEVs was titrated (Additional file 3: Fig. S2).

**Fig. 5:**
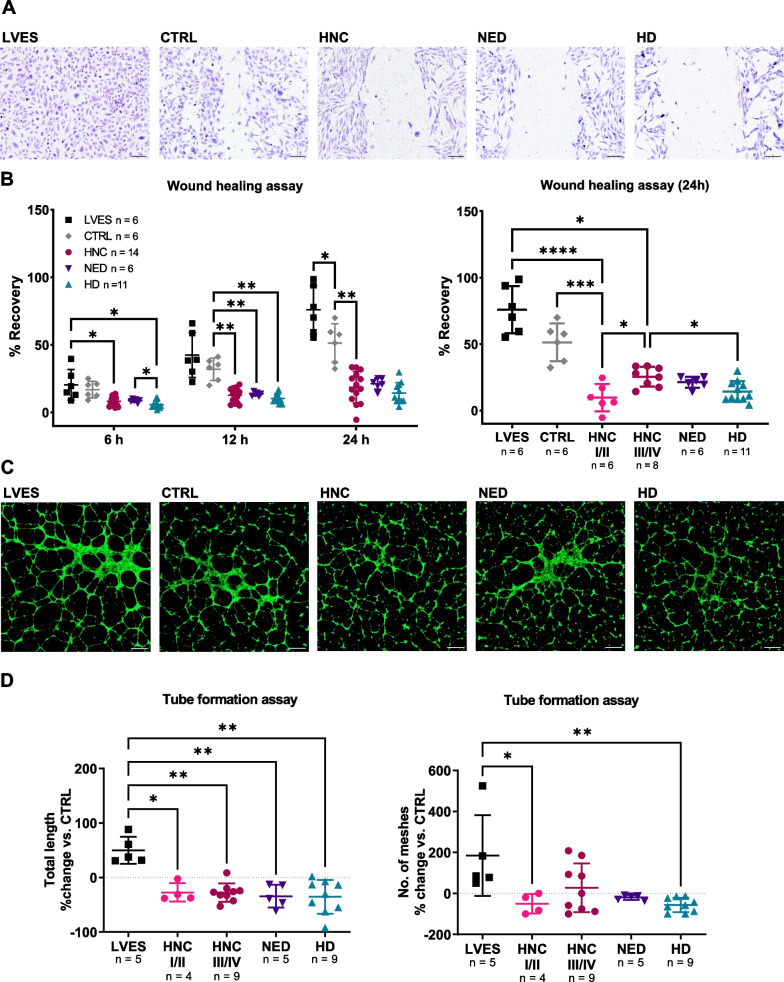
Functional wound healing and tube formation assays of HUVECs co-incubated with sEVs. HUVECs were grown to confluence and mechanically wounded to detect the recovered area in the presence of sEVs. **A** Representative images of migration assays after 24 h of co-incubation with sEVs are visualized by inverted microscopy (magnification = 10x, scale = 150 µm). **B** The scatter plots present the recovered area in percent of the measured gap after 24 h from scratching. **C** Representative fluorescence microscopy images of tube formation assays after 8 h co-incubation of HUVECs and sEVs. LVES-containing medium was used as a positive control. Magnification = 2.5x, scale = 200 µm D Scatter plots display the modification of total tube length and number of meshes compared to the PBS control (dotted line). *p < 0.05; **p < 0.01; ***p < 0.001; ****p < 0.0001

Interestingly, crystal violet staining showed that after incubation with sEVs (Fig. 4), ECs underwent a morphological change, in which the cells elongated and partially detached, whereas in the control images (PBS or LVES), the cells retained their cobblestone-like arrangement. Next, to investigate the impact of sEVs on the wound healing capacity of HUVEC, a confluent monolayer of cells was wounded mechanically and the regeneration of the cell layer was monitored (Fig. 5A, B). After 24 h, the regeneration of the cell layer was induced by LVES and plasma sEVs, but not by the PBS control (CTRL). The reduction of wound healing by plasma sEVs correlated with the disease activity of the HNC patients or HDs, i.e. sEVs from HDs, NED and early-stage HNC patients showed a stronger inhibition than sEVs from advanced-stage HNC patients.

Furthermore, tube formation assays were performed to examine the effect of sEVs on the tubulogenesis of HUVECs. HUVECs were seeded on a basement membrane matrix, treated with sEVs and tube formation was monitored every 2 h (Fig. 5C, D). Overall, ECs treated with sEVs formed smaller and less defined networks. As expected, the LVES-containing medium increased the total length of tubules, whereas sEVs from HNC, NED and HD plasma reduced them. Of note, late-stage HNC sEVs appeared to increase the number of developed meshes compared to the other groups.

**Fig. 6:**
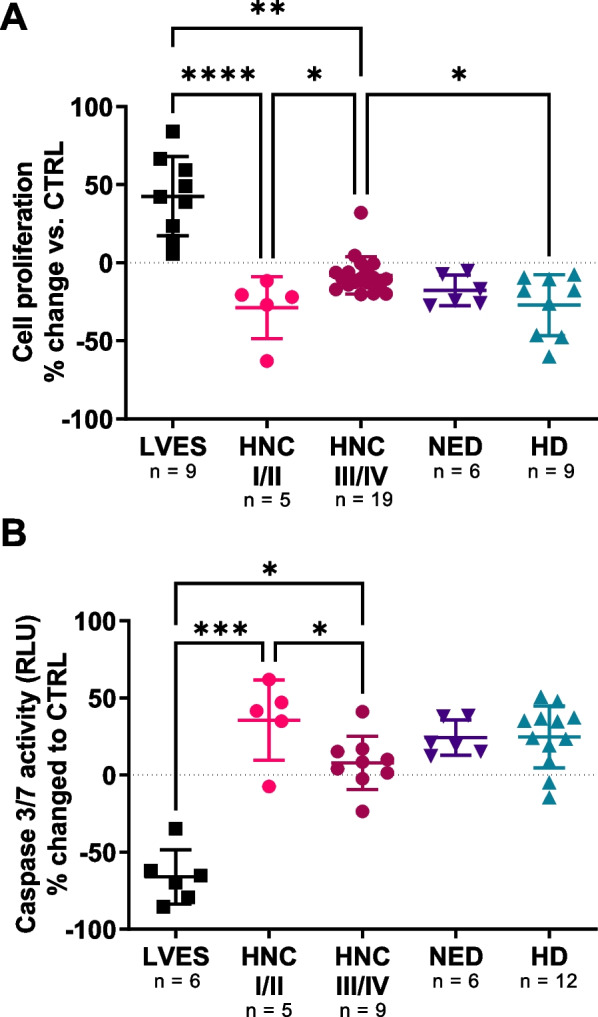
Proliferation and apoptosis of HUVECs in the presence of plasma sEVs. HUVECs and plasma sEVs from HNC patients, NED and HDs were co-incubated for 24 h. Proliferation (MTS assay) and apoptosis induction (Caspase3/7) of HUVECs was detected and visualized by scatter plots. The percentage changes compared to the PBS control (dotted line) after stimulation for 24 h is shown. The plots of the **A** MTS Assay and **B** Caspase-Glo 3/7 assay show the inhibition of proliferation and apoptosis induction by sEVs in relation to the disease activity and tumor stage of the HNC patients. *p < 0.05; **p < 0.01; ***p < 0.001; ****p < 0.0001

Next, we assessed the EV-mediated effect on HUVEC proliferation. To this end, sEVs, LVES-containing medium or PBS (CTRL) were added and the proliferation was assessed after 24 h using colorimetric MTS assays. The assay (Fig. 6A) shows that the relative proliferation of ECs was inhibited most by HD, NED and HNC stage I/II sEVs, while the suppression by sEVs from advanced-stage HNCs (UICC III/IV) was less.

Since cell detachment was observed after treatment in previous assays, apoptosis of endothelial cells was investigated by measuring caspase 3/7 activity (Fig. 6B). Caspase 3/7 was induced in HUVECs co-incubated with sEVs from HD, NED and early stage HNCs (UICC I/II), while sEVs from advanced stage HNCs did not induce apoptosis of HUVECs (comparable to CTRL/PBS).

All in all, in this manuscript we have found that plasma sEVs inhibit proliferation, migration and tube formation and promote apoptosis of endothelial cells. However, this adverse effect decreases with disease activity and higher tumor stages. Other clinical factors like HPV status, tumor site as well as gender and age had no influence on the angiogenic effect of sEVs on HUVECs (Additional file 4: Fig. S3, Additional file 5: Fig. S4, Additional file 6: Fig. S5, Additional file 7: Fig. S6).

## Discussion

A sufficient blood supply is indispensable for cancer growth, progression, and metastasis, and as such defines angiogenesis as one of the hallmarks of cancer (Hanahan and Weinberg 2011). While angiogenesis mediated by TEV from HNC cell lines has been reported to have strong pro-angiogenic potential (Ludwig et al. 2018, 2020a), the angiogenic properties of plasma sEVs from HNC patients have not been examined in detail. Our data indicate that plasma sEVs from HDs have strong anti-angiogenic properties. Compared with HD sEVs, sEVs from NED or HNC patients have pro-angiogenic activities that increase with higher disease activity and advanced tumor stages.

A closer examination of the sEV protein profile reveals mainly the abundance of anti-angiogenic proteins in sEVs from both: HNC patients and HDs. The anti-angiogenic thrombospondin-1 is known to inhibit migration, proliferation, survival, and to promote apoptosis of ECs by directly binding to CD36 and antagonizing the activity of VEGF (Lawler and Lawler 2012). However, recently Liu et al. showed that thrombospondin-1 induced PD-L1 mediated immunosuppression in osteosarcoma resulting in increased tumor growth (Liu et al. 2022), a mechanism that has been described for HNC as well (Ludwig et al. 2017). Besides, platelet factor 4 and serpin F1 have strong anti-angiogenic functions mainly through inhibition of VEGF receptor signaling (Pilatova et al. 2013; Liu et al. 2005; Dawson et al. 1999). Thus, the observed anti-angiogenic effect of plasma sEVs on ECs is not surprising. Interestingly, in sEVs from HNC patients, the level of pro-angiogenic MMP-9 is increased in comparison to HD. Notari et al. reported that MMP-9 degrades the extracellular matrix (ECM) and basement membrane and activates cytokines promoting angiogenesis, invasion and metastasis by induction of VEGF (Bergers et al. 2000). Additionally, during hypoxia, MMP-9 proteolytically degrades the anti-angiogenic serpin F1 (Notari et al. 2005). MMP-9 is an unfavorable prognostic factor in HNC (Vicente et al. 2005; Liu et al. 2010), bladder cancer (Miao et al. 2017) and ovarian cancer (Jia et al. 2017), which might indicate its key role in angiogenesis.

The internalization of sEVs by ECs and other target cells mediates angiogenesis and immunosuppression in carcinogenesis (Whiteside 2016). Here, we observed the internalization of PKH-dyed sEVs by ECs after 4 h. PKH-dye has been reported to bind non-specifically to lipoproteins (Ludwig et al. 2020a; Lawler and Lawler 2012), our study is supported by others confirming the uptake of PKH-labeled HNC TEV by ECs mainly via receptor-mediated endocytosis after 2–4 h (Ludwig et al. 2018; Pužar Dominkuš et al. 2018).

Crystal violet staining showed a change in cell morphology with elongated fibroblast-like cells and increased cell detachment, which may indicate changes in function and promotion of apoptosis caused by sEV stimulation. The spindle-shaped morphology could also point to an endothelial-to-mesenchymal transition (EndMT), which has been reported for melanoma-derived sEVs (Yeon et al. 2018) and also as a result of MMP-9 stimulation within the endothelial cells (Zhao et al. 2016). Interestingly, the functional effect of sEVs on ECs was stage-dependent: Advanced-stage sEVs induced a better wound recovery, less suppression of proliferation and reduced apoptosis of ECs than sEVs from early-stage HNC, NED or HD. However, the functional impact of sEVs on tubulogenesis is controversial: Some advanced-stage HNC sEVs increased the number of meshes compared to the control but not the total length of the tubules, while HD sEVs showed the strongest inhibition of tube formation. Endothelial cell migration and proliferation are essential for angiogenesis and are regulated by cytokines such as VEGF, bFGF, angiopoietins, and angiogenin, some of which are also found in sEV preparations (Lamalice et al. 2007). Tubulogenesis is a complex process involving migration, proliferation, lumen formation and sprouting of ECs (Geudens and Gerhardt 2011), but without stabilization, the immature capillaries rapidly become apoptotic and regress (Benjamin et al. 1998). Here, plasma sEVs seem to affect tubulogenesis already in the growth phase. In vitro, MMP-9 expression was crucial for cell migration and tube formation in microvascular ECs (Jadhav et al. 2004) and MMP-9 treatment substantially increased tube formation in HUVECs (Santhekadur et al. 2012). Therefore, the elevated levels of MMP-9 in HNC sEVs and internalization by ECs might contribute to the less pronounced anti-angiogenic activity, while the high levels of thrombospondin-1 in sEV preparations might be responsible for the increased apoptosis.

Recently, studies have also pointed to a central role of immunosuppressive adenosine in tumor angiogenesis (Ludwig et al. 2020a, 2020b). Its production is stimulated by increased CD39 and CD73 cargo on HNC cell line sEVs, but also on plasma-derived sEVs, possibly contributing to their effect on ECs (Ludwig et al. 2017).

To date, most functional studies have focused on the role of sEVs derived from tumor cell lines showing a pro-angiogenic effect on endothelial cells (i.e. glioblastoma (Skog et al. 2008; Lang et al. 2017), breast cancer (Maji et al. 2017; Eichelser et al. 2014) and colorectal cancer (Huang and Feng 2017) but also HNC (Ludwig et al. 2018)). Of note, sEVs are secreted by all cell types. Consequently, plasma contains a mixture of sEVs from various cells, including immune cells, platelets, erythrocytes and ECs. Some of these cells secrete sEVs with pro- or anti-angiogenic functions depending on the stimulus for vesicle release (Todorova et al. 2017; Yang et al. 2008; Ramakrishnan et al. 2016). There have been some controversial studies on sEVs derived from serum of cancer patients that promoted angiogenesis and showed no inhibitory effect on endothelial cells (Hsu et al. 2017; O'Brien et al. 2013). However, the use of serum as an sEV source leads to a higher amount of platelet-associated-proteins and EVs (Zhang et al. 2012) that were previously shown to also have pro-angiogenic properties and might limit these results (Kim et al. 2004).

Possibly, advanced HNC might secrete elevated levels of tumor-derived sEVs that stimulate angiogenesis in comparison to HD and NED, in which anti-angiogenic sEVs prevail.

To verify the hypothesis of a stronger pro-angiogenic effect of TEVs within HNC patients’ sEVs, bead-based separation techniques of tumor-derived sEVs from plasma as described by others could be employed for future studies (Beccard et al. 2020; Theodoraki et al. 2018; Benecke et al. 2022) and would provide a deeper insight into the angiogenic potential of TEVs and sEVs in general.

The preparation of sEVs by size exclusion chromatography provides a high yield of high-quality vesicles (Hong et al. 2016). However, functional analyses of plasma sEVs requires a fresh preparation of the specimens to provide reliable results (Muller et al. 2014). In some experiments within this study, the performance of the experiments required high protein amounts and several repetitions to show reproducible and reliable results, which resulted in varying numbers of patients and HD in the different experiments.

Nevertheless, the following conclusions can be drawn from this study: In summary, plasma sEVs from HNC patients modulate angiogenesis in a stage-dependent manner. Since the greatest functional differences between HD and HNC are observed at advanced stages, analysis of the signaling pathways and proteomic profiles responsible for these differences could lead to the discovery of novel prognostic biomarkers for HNC. Because the clinical utility of angiogenesis inhibitors in the treatment of HNC patients remains unclear and some are associated with increased toxicity (e.g. bevacizumab) (Argiris et al. 2019), targeting sEV uptake by recipient endothelial cells or sEV release by tumor cells may represent an alternative option. Further studies are needed to expand the understanding of sEVs in angiogenesis.

## Conclusions

The pro-angiogenic properties of plasma sEVs are increasing with higher disease activity and tumor stages, which might reflect a higher abundance of tumor-derived sEVs (TEV) in patients with advanced-stage HNC. Ultimately, targeting TEV and sEV-mediated angiogenesis might serve as a promising tool for cancer therapy in the future.

## Supplementary Information

**Additional file 1: Table S1.** Mean pixel density of spots on Antibody Array.

**Additional file 2. Fig. S1.** Original Western blot images for sEV characterization.

**Additional file 3. Fig. S2.** Titration curve for optimal sEV concentration used for the MTS assay.

**Additional file 4. Fig. S3.** Correlation of clinical factors with the wound healing effect of sEVs.

**Additional file 5. Fig. S4.** Correlation of clinical factors with the effect of sEVs on tubulogenesis.

**Additional file 6. Fig. S5.** Correlation of clinical factors with the effect of sEVs on EC proliferation.

**Additional file 7. Fig. S6.** Correlation of clinical factors with apoptosis induction of sEVs.

## Data Availability

The participants of this study did not give written consent for their data to be shared publicly, so due to the sensitive nature of the research supporting data is not available.

## Abbreviations

ApoA1: Apolipoprotein A1
BCA: Bicinchoninic acid
bFGF: Basic fibroblast growth factor
BSA: Bovine serum albumin
DAPI: 4',6-Diamidino-2-phenylindol
DPPIV: Dipeptidyl peptidase IV
EC: Endothelial cell
ECM: Extracellular matrix
EGF: Epidermal growth factor
EV: Extracellular vesicle
FGF: Fibroblast growth factor
Grp94: Glucose-regulated protein 94
HD: Healthy donor
HNC: Head and neck cancer
HNSCC: Head and neck squamous cell carcinoma
HRP: Horseradish peroxidase
HUVEC: Human umbilical vein endothelial cells
IGFBP: Insulin-like growth factor-binding protein
IL-8: Interleukin 8
LDEV: Lactate dehydrogenase elevating virus
LVES: Larger vessel endothelial supplement
MMP-8: Matrixmetalloproteinase 8
MMP-9: Matrixmetalloproteinase 9
MTS: (3-(4,5-Dimethylthiazol-2-yl)-5-(3-carboxymethoxyphenyl)-2-(4-sulfophenyl)-2H-tetrazolium)
NED: No evident disease
NTA: Nanoparticle tracking analysis
PBS: Phosphate buffered saline
PD-1: Programmed cell death protein 1
PDGF: Platelet-derived growth factor
RT: Room temperature
sEV: Small extracellular vesicle
TEM: Transmission electron microscopy
TEV: Tumor-derived sEVs
TGF-β: Transforming growth factor β
TME: Tumor microenvironment
TSG101: Tumor susceptibility gene 101 protein
UICC: Union for International Cancer Control
uPA: Urokinase-type Plasminogen Activator
VEGF: Vascular Endothelial Growth Factor
