# Supplementary material for: Plasma-derived small extracellular vesicles unleash the angiogenic potential in head and neck cancer patients
Source: Mol Med. 2023 May 24;29:69. doi: 10.1186/s10020-023-00659-w (PMC10207688; doi:10.1186/s10020-023-00659-w)
Supplement: Supplementary file 1 — Additional file 1: Table S1. Mean pixel density of spots on Antibody Array. [file 10020_2023_659_MOESM1_ESM.docx]

| Additional file 1: Table S1. Mean pixel density of spots on Antibody Array | | |
| --- | --- | --- |
| **Angiogenic factors** | **HNC** | **HD** |
| Angiogenin (1) | 2657 | 4770 |
| IGFBP-1 (4) | 1218 | 2238 |
| IGFBP-3 (5) | 1476 | 2281 |
| MMP-8 (6) | 1734 | 3075 |
| MMP-9 (7) | 8133 | 2031 |
| uPA (14) | 1157 | 1764 |
| Angiopoetin-2 (2) | 5277 | 4953 |
| DPPIV (3) | 32404 | 19874 |
| Pentraxin 3 (8) | 22675 | 11956 |
| Serpin E1 (10) | 33940 | 21579 |
| Platelet Factor 4 (9) | 160071 | 132936 |
| Serpin F1 (11) | 87339 | 45922 |
| Thrombospondin-1 (12) | 187015 | 171131 |
| Thrombospondin-2 (13) | 6003 | 3164 |
