# Supplementary figures and images for: Plasma-derived small extracellular vesicles unleash the angiogenic potential in head and neck cancer patients
Source: Mol Med. 2023 May 24;29:69. doi: 10.1186/s10020-023-00659-w (PMC10207688; doi:10.1186/s10020-023-00659-w)

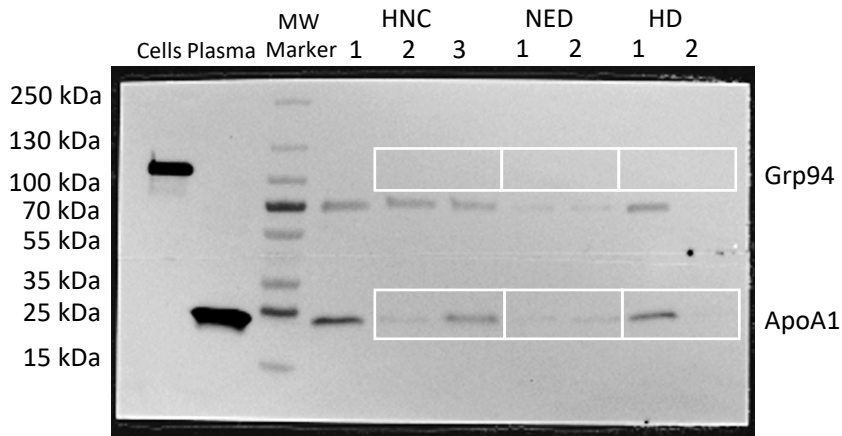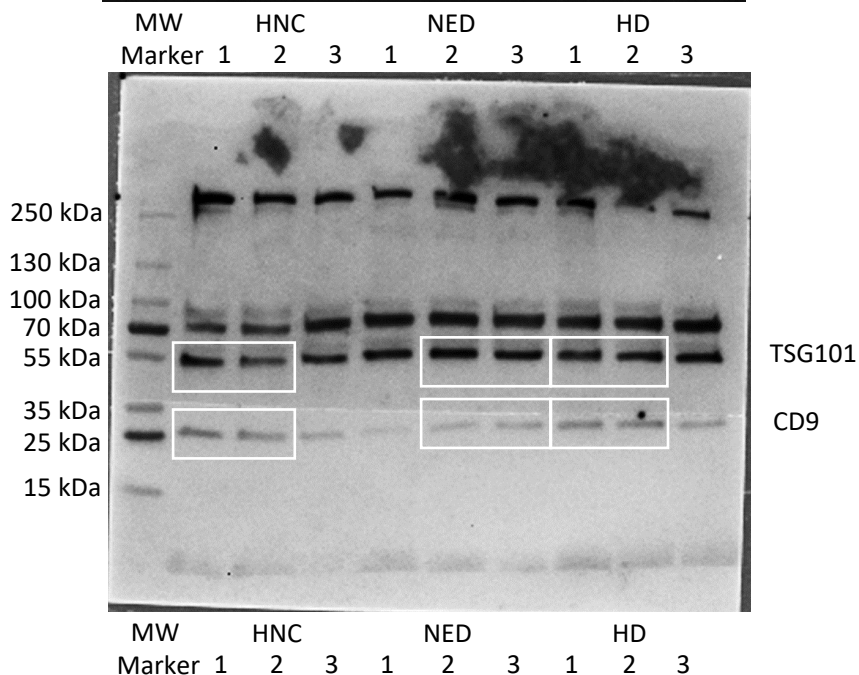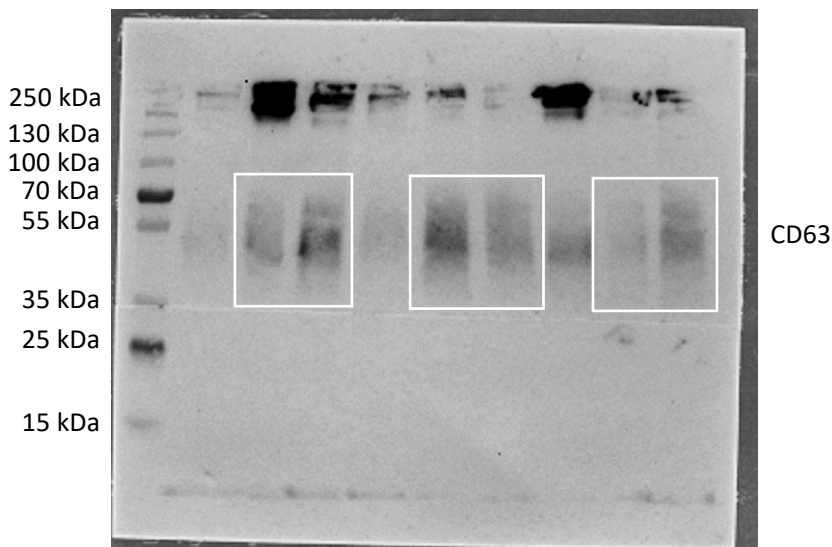

Supplement: Supplementary file 2 — Additional file 2. Fig. S1. Original Western blot images for sEV characterization. [file 10020_2023_659_MOESM2_ESM.pdf]

## MTS Assay

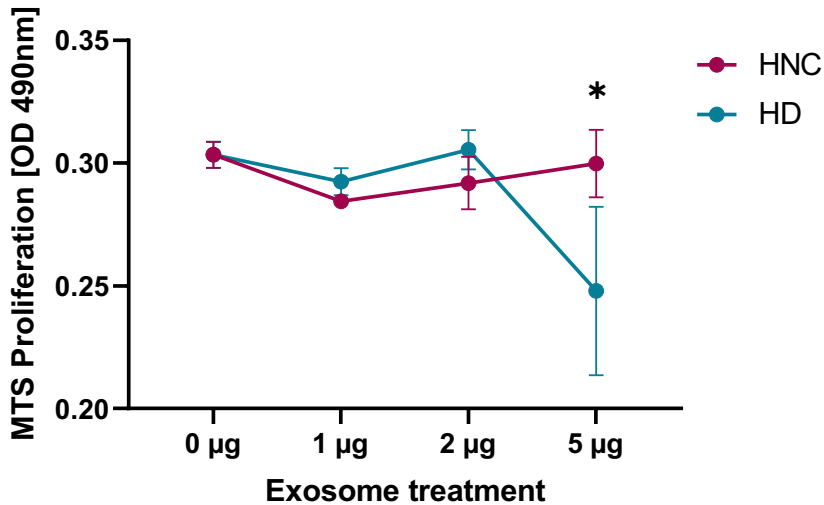

Supplement: Supplementary file 3 — Additional file 3. Fig. S2. Titration curve for optimal sEV concentration used for the MTS assay. [file 10020_2023_659_MOESM3_ESM.pdf]

# MTS Proliferation Assay

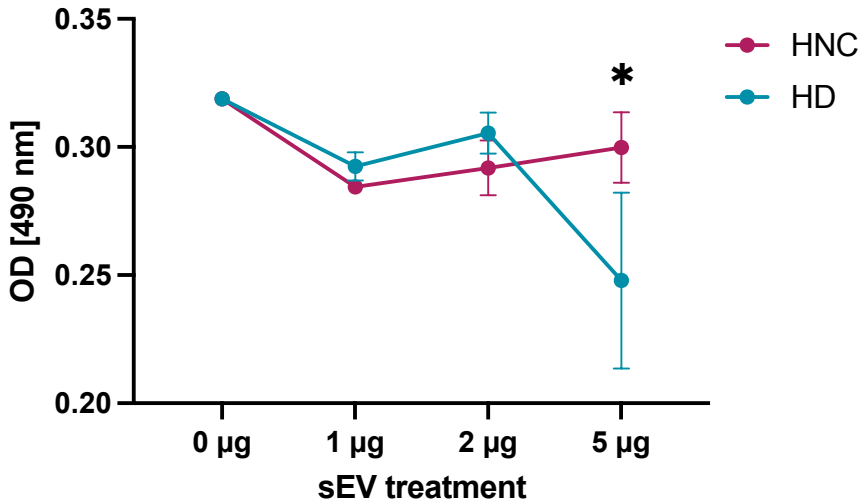

Supplement: Supplementary file 4 — Additional file 4. Fig. S3. Correlation of clinical factors with the wound healing effect of sEVs. [file 10020_2023_659_MOESM4_ESM.pdf]

# MTS proliferation assay

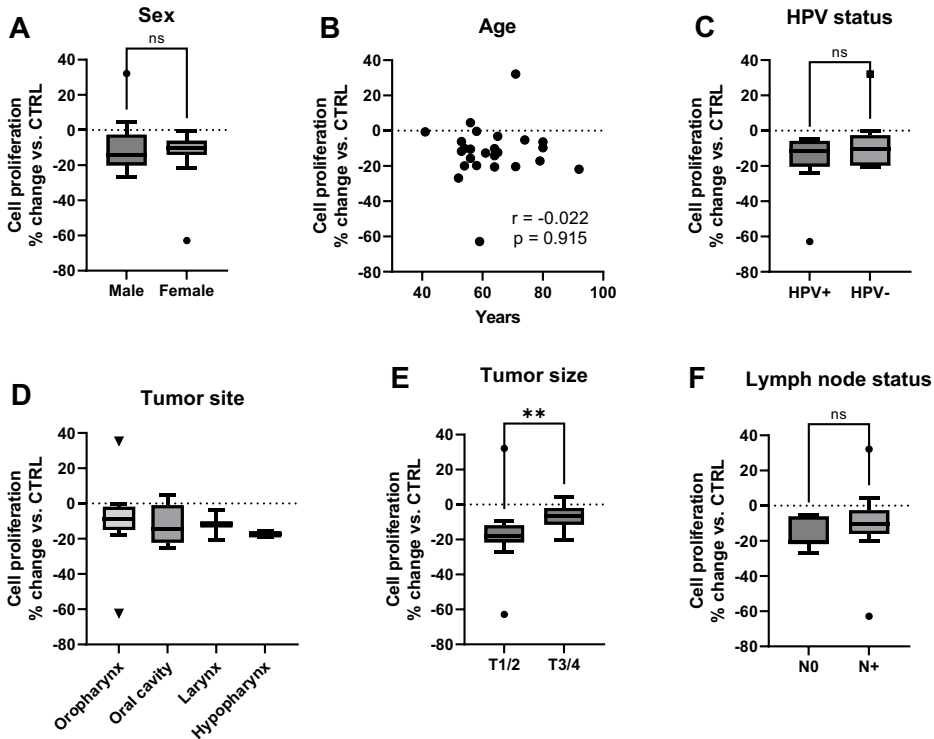

Supplement: Supplementary file 6 — Additional file 6. Fig. S5. Correlation of clinical factors with the effect of sEVs on EC proliferation. [file 10020_2023_659_MOESM6_ESM.pdf]

# Caspase 3/7 Assay

**A****Sex**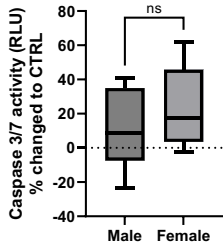**B****Age**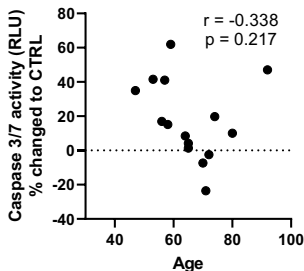**C****HPV status**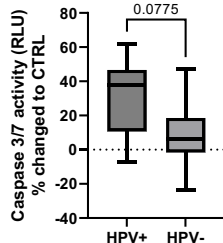**D****Tumor site**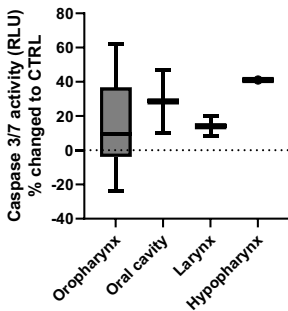**E****Tumor size**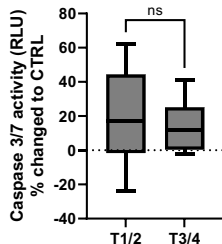**F****Lymph node status**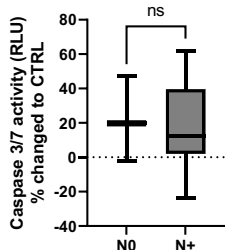

Supplement: Supplementary file 7 — Additional file 7. Fig. S6. Correlation of clinical factors with apoptosis induction of sEVs. [file 10020_2023_659_MOESM7_ESM.pdf]
