# Supplementary material for: Plasma-derived small extracellular vesicles unleash the angiogenic potential in head and neck cancer patients
Source: Mol Med. 2023 May 24;29:69. doi: 10.1186/s10020-023-00659-w (PMC10207688; doi:10.1186/s10020-023-00659-w)

Tube formation assay

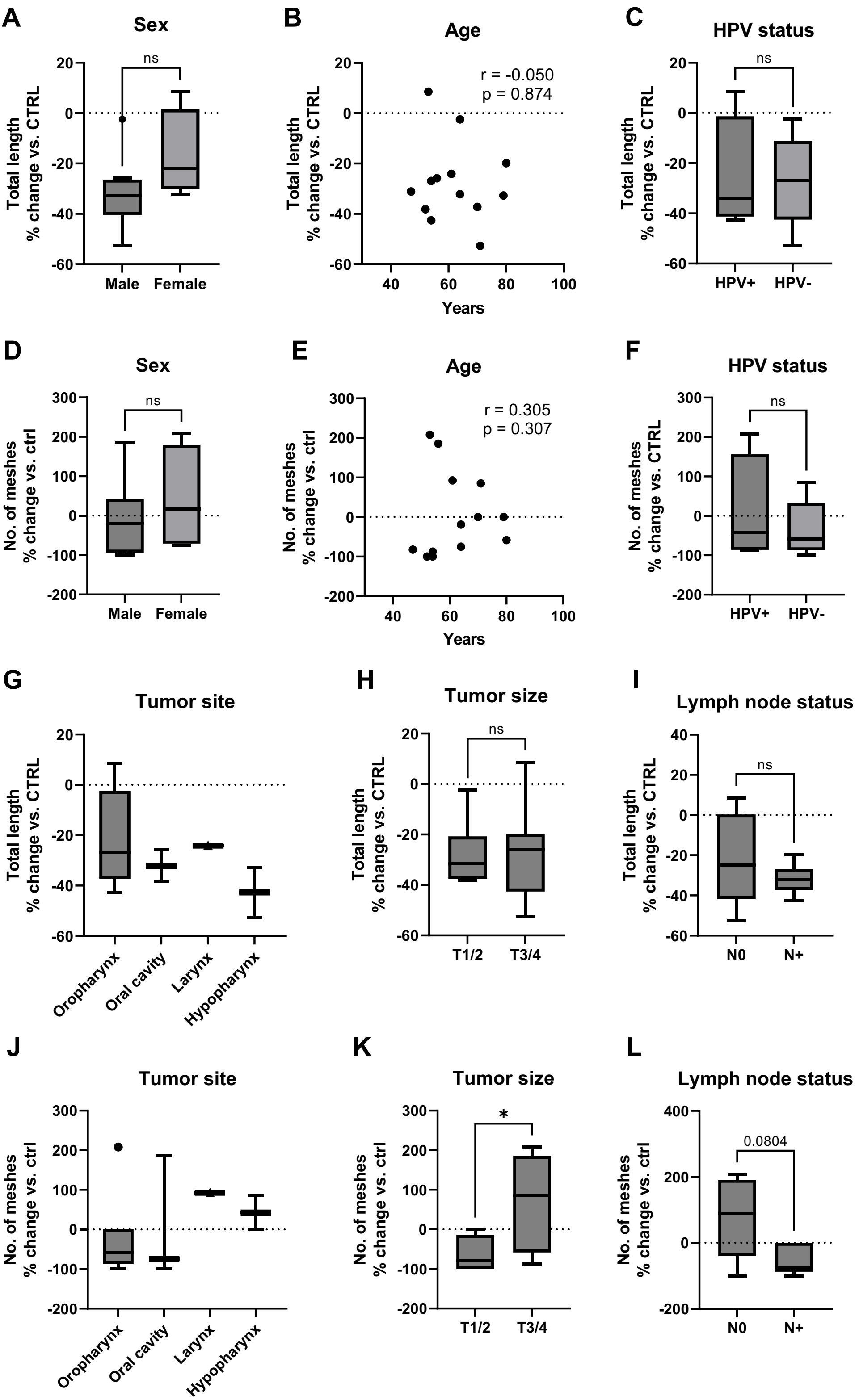

Supplement: Supplementary file 5 — Additional file 5. Fig. S4. Correlation of clinical factors with the effect of sEVs on tubulogenesis. [file 10020_2023_659_MOESM5_ESM.pdf]
